# Identifying prognostic factors of severe metabolic acidosis and uraemia in African children with severe falciparum malaria: a secondary analysis of a randomized trial

**DOI:** 10.1186/s12936-021-03785-0

**Published:** 2021-06-25

**Authors:** Grace Mzumara, Stije Leopold, Kevin Marsh, Arjen Dondorp, Eric O. Ohuma, Mavuto Mukaka

**Affiliations:** 1 Malawi Liverpool Wellcome Trust, Queen Elizabeth Central Hospital College of Medicine, Chichiri 3, P.O. Box 30096, Blantyre, Malawi; 2 University of Malawi, College of Medicine, Blantyre, Malawi; 3 University of Oxford, Oxford, UK; 4 Centre for Tropical Medicine and Global Health, Oxford, UK; 5 KEMRI-Wellcome Trust Research Programme, Kilifi, Kenya; 6 Mahidol-Oxford Tropical Medicine Research Unit, Bangkok, Thailand; 7 Maternal, Adolescent, Reproductive and Child Health (MARCH) Centre, School of Hygiene and Tropical Medicine (LSHTM), London, UK

**Keywords:** Severe malaria, Metabolic acidosis, Acute kidney injury, Plasmodium falciparum, Africa

## Abstract

**Background:**

Severe metabolic acidosis and acute kidney injury are major causes of mortality in children with severe malaria but are often underdiagnosed in low resource settings.

**Methods:**

A retrospective analysis of the ‘Artesunate *versus* quinine in the treatment of severe falciparum malaria in African children’ (AQUAMAT) trial was conducted to identify clinical features of severe metabolic acidosis and uraemia in 5425 children from nine African countries. Separate models were fitted for uraemia and severe metabolic acidosis. Separate univariable and multivariable logistic regression were performed to identify prognostic factors for severe metabolic acidosis and uraemia. Both analyses adjusted for the trial arm. A forward selection approach was used for model building of the logistic models and a threshold of 5% statistical significance was used for inclusion of variables into the final logistic model. Model performance was assessed through calibration, discrimination, and internal validation with bootstrapping.

**Results:**

There were 2296 children identified with severe metabolic acidosis and 1110 with uraemia. Prognostic features of severe metabolic acidosis among them were deep breathing (OR: 3.94, CI 2.51–6.2), hypoglycaemia (OR: 5.16, CI 2.74–9.75), coma (OR: 1.72 CI 1.17–2.51), respiratory distress (OR: 1.46, CI 1.02–2.1) and prostration (OR: 1.88 CI 1.35–2.59). Features associated with uraemia were coma (3.18, CI 2.36–4.27), Prostration (OR: 1.78 CI 1.37–2.30), decompensated shock (OR: 1.89, CI 1.31–2.74), black water fever (CI 1.58. CI 1.09–2.27), jaundice (OR: 3.46 CI 2.21–5.43), severe anaemia (OR: 1.77, CI 1.36–2.29) and hypoglycaemia (OR: 2.77, CI 2.22–3.46)

**Conclusion:**

Clinical and laboratory parameters representing contributors and consequences of severe metabolic acidosis and uraemia were independently associated with these outcomes. The model can be useful for identifying patients at high risk of these complications where laboratory assessments are not routinely available.

## Background

A major challenge to preventing mortality from malaria is early identification and mitigation of clinical states predictive of death from malaria. The World Health Organization (WHO) Africa region bears the brunt as malaria mortality accounts for 86% of global malaria cases, and 80% of all malaria related deaths [1]. Despite prompt administration of anti-malarials, 5.8%–24% of childhood deaths from severe malaria occur on the first day of admission [2, 3]. Metabolic acidosis and acute kidney injury are two of three (in addition to coma) complications of malaria most predictive for a fatal outcome of severe malaria [2, 4–10]. However, diagnosis of metabolic acidosis or acute kidney injury requires laboratory facilities beyond the basic test facilities often available. Assessing the base excess to quantify metabolic acidosis, requires a blood gas machine, and assessing plasma lactate, which can be a surrogate maker for acidosis, is often not routinely available. Acute kidney injury can be identified rapidly if the patient with severe malaria is anuric. However, up to 80% of severe malaria patients with acute kidney injury (AKI) are non-oliguric [11–13]. Identifying these children with a high risk of having AKI could be beneficial in resource-poor settings.

In children with severe malaria, metabolic acidosis is present in up to 50% of cases, and is strongly associated with a fatal outcome [10, 14, 15]. Metabolic acidosis in malaria is mainly a result of increased lactate (LA) production from anaerobic glycolysis and to a smaller extent from *Plasmodium falciparum* parasites [5, 8]. The plasma and urinary acids α-hydroxybutyric acid (αHBA), β-hydroxybutyric acid (βHBA), and p-hydroxyphenyllactic acid (HPLA), also contribute to metabolic acidosis [5–7, 16]. Failure of hepatic gluconeogenesis and lack of kidney clearance due to acute kidney injury commonly compound this problem [8, 16]. These biochemical changes cause physiologic responses and clinical signs that can be used to identify metabolic acidosis in patients.

The clinical signs associated with metabolic acidosis are varied and there is need for reliable criteria to make this diagnosis clinically. Although deep breathing is a sign of respiratory distress most commonly described in relation to metabolic acidosis, its association has been contested in several studies [2, 14, 17, 18]. Similarly, there is varied significance in the association of metabolic acidosis and the presence of coma or convulsions [17, 19]. Due to the overlapping nature of these and other clinical signs, it is challenging to identify metabolic acidosis clinically [20].

In children, acute kidney injury is underdiagnosed and presents a growing concern for kidney disease as a global health problem in Africa [21]. In Nigeria, malaria was the third commonest cause of AKI among children admitted to a nephrology unit [13]. Among Congolese children in intensive care, black water fever from malaria was the predominant cause of AKI [12]. In Uganda, nearly half (45.5%) of children with severe malaria had kidney disease improving global outcomes defined AKI [21]. Challenges in diagnosing AKI in resource limited settings mean that it is underdiagnosed consequently undertreated, leading to increasing mortality and an increased risk of chronic kidney injury and neurocognitive disorders [4, 10, 21–23].

Having reliable surrogate markers that identify metabolic acidosis and kidney injury could improve their diagnosis. However, studies on clinical signs of severe metabolic acidosis and AKI in malaria often have low sensitivity and high inter-observer variability [18, 20, 24]. Reliably identifying these complications of malaria would improve the efficiency of therapeutic approaches within the first 24 h of admission.

The aim of this study was to develop prognostic models for identifying factors related to severe metabolic acidosis and kidney injury using clinical signs among children admitted with severe malaria.

## Methods

### Study design, study population and sampling

This is a secondary analysis of the open label 'Artesunate *versus* quinine in the treatment of severe falciparum malaria in African children' randomized controlled trial (AQUAMAT) in eleven centres of nine African countries [25]. The main aim of the study was to compare mortality outcomes in the quinine and artesunate arms of the trial. Selected patients had a positive blood antigen dipstick test for *Plasmodium falciparum* histidine rich protein2, and a physician’s clinical diagnosis of severe malaria. Severe malaria was defined as patients with a positive malaria test and at least one of the WHO symptoms [25]. Patients with previous treatment of quinine (40 mg/kg on first day and 30 mg/kg on any subsequent day) or artesunate derivatives within 24 h preceding admission to the study were excluded [25].

The AQUAMAT randomized trial enrolled 5425 children from 11 centres in nine African countries between October 2005 and July 2010. The participating countries and their number of participants are: The Gambia (502, 9.3%), Ghana (436, 8%), Kenya (442, 8.3%), Tanzania (1461, 26.9%), Nigeria (450, 8.3%), Uganda (663, 12.2%), Mozambique (664, 12.2%), Rwanda (386, 7.1%), and Democratic Republic of the Congo (422, 7.8%) [25]. There were 2713 patients in the artesunate arm and 2713 patients in the quinine arm analysed by intention to treat [25]. Overall mortality was 9.7% (527), with 230 (8.5%) deaths in the artesunate group and 297 (10.9%) deaths in the quinine group [25]. The study supported adopting artesunate as the first-line of treatment for *P. falciparum* malaria in children. Other analyses of mortality findings have been discussed in secondary analyses of the AQUAMAT study [4, 25].

### Definitions of model outcomes and candidate predictors

**Fig. 1:**
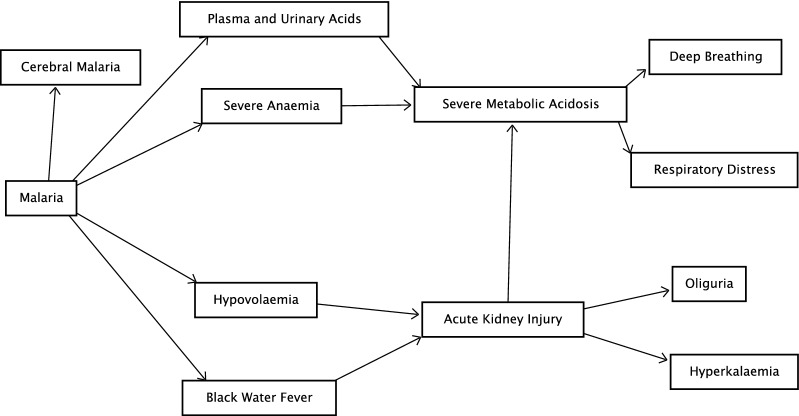
Conceptual framework of features associated with severe metabolic acidosis and acute kidney injury in children with severe falciparum malaria [31]

Literature on severe metabolic acidosis and AKI in children with severe malaria were searched to identify features associated with these complications that were studied or previously described (Fig. 1). Candidate predictors that were complications of malaria and those that either caused or resulted from severe metabolic acidosis or AKI were included. For severe metabolic acidosis, this includes contributing factors like anaemia and consequences of metabolic acidosis, like deep breathing [17, 26]. Similarly, for AKI candidate predictors include causative features like black water fever and hypovolaemia [27, 28].

The outcome and candidate predictor cut-off points were selected according to WHO classifications for severe metabolic acidosis and kidney injury in children with severe malaria [29]. Accordingly, severe metabolic acidosis was defined as a base excess of less than 8 mmol/l [29]. This cut-off for base excess has also been used in studies in similar settings [14, 20]. Kidney injury was assessed using uraemia, defined as a blood urea nitrogen (BUN) of more than 20 mg/dl [29]. Candidate predictors for each outcome were selected based on WHO signs of severe malaria and variables described in literature to be associated with each outcome. Ideally, AKI should have been assessed and staged by kidney disease: improving global outcomes (KDIGO) criteria, using serum creatinine and urine output [30]. Only blood urea nitrogen was collected in this study, thus limiting the assessment to uraemia.

### Definitions of parameters

Severe metabolic acidosis: Base Excess <  − 8  mmol/l.

- Uraemia: Blood Urea Nitrogen > 20 mg/dl.
- Severe anaemia: Hb < 5 g/dl.
- Mild anaemia: Hb > 5, < 10 g/dl.

### Statistical analysis

The forward selection method was used for inclusion of predictors for the diagnosis of severe metabolic acidosis and uraemia. To select candidate predictors for each outcome, a univariable logistic regression was used and a 5% significance level threshold for inclusion of variables into the model using forward selection approach.

Key predictors and some selected patient demographics were included in the multivariable logistic regression analysis irrespective of statistical significance. The multivariable model was adjusted for trial arm (quinine and artesunate), patient demographics (including country of origin) and, included only clinical variables that were statistically significant.

The performance of the predictive models was assessed through calibration and discrimination. A calibration plot of predicted observed against predicted events was plotted for 10 equal groups to measure the agreement in probabilities of developing the outcomes. Model discrimination was assessed by area under the curve (AUC) of a receiver operating characteristic (ROC). Using the sampling with replacement method, 5000 simulations of the model were computed to assess the generalizability of the models by comparing the bootstrapped c-index and confidence interval to the AUC obtained from the logistic model.

This data was analysed using Stata software package version 14.2, StataCorp, College Station, TX: StataCorp LP.

### Ethical considerations

Ethical approval for the AQUAMAT study, registered under ISRCTN50258054, was obtained from each participating institutional or national ethics committee in addition to the Oxford Tropical Research Ethics committee [25]. The use of data for this secondary analysis was approved by the Oxford-Mahidol research Unit Data Access Committee through an application for ‘Datasets under the Custodianship of Mahidol Oxford Tropical Medicine Research Unit (MORU) Tropical Network’. The database used for this analysis was de-identified and anonymized.

## Results

The baseline characteristics (Table 1) were collected at admission and there were no significant differences between the artesunate and quinine group.

**Table 1:** Baseline characteristics of patients admitted to the AQUAMAT trial

Variable	N (%)	Artesunate N (%)	Quinine N (%)
Sample size: 5425			
Patient demographics			
Congo	422 (7.8)	212 (50.2)	210 (49.8)
Gambia	502 (9.3)	250 (49.8)	252 (50.2)
Ghana	436 (8)	218 (50)	218 (50)
Kenya	442 (8.3)	219 (49.6)	223 (50.5)
Mozambique	664 (12.2)	332 (50)	332 (50)
Nigeria	450 (8.3)	226 (50.2)	224 (49.8)
Rwanda	386 (7.1)	194 (50.3)	192 (49.7)
Tanzania	1461 (26.9)	729 (49.9)	732 (50.1)
Uganda	663 (12.2)	333 (50.2)	330 (49.8)
Age (Median, IQR: 25–75)	2 (1- 4)	2 (1–4)	2 (1−4)
Females	2611 (48.1)	1298 (47.7)	1316 (48.5)
Male	2815 (51.9)	1418 (52.3)	1397 (51.5)
Clinical characteristics			
Coma at admission (BCS < = 2)	1823 (33.6)	881 (48.3)	942 (51.7)
Severe prostration (Not able to breast feed < 6 m or able to sit > 6 m)	2974 (54.8)	1505 (50.6)	1469 (49.3)
Convulsions (> 30 min)	1355 (31.2)	637 (47)	718 (52.9)
Decompensated shock (BP < 70mmhg)	178 (3.3)	88 (49.4)	90 (50.6)
Deep breathing	938 (17.3)	495 (52.8)	443(47.2)
Respiratory distress (costal in drawing/ recession / respiratory insufficiency	867 (15.9)	439 (50.6)	428 (49.4)
Symptomatic severe anaemia (severe pallor combined with respiratory distress)	2213 (40.8)	1131 (51.1)	1082 (48.9)
Black water fever (haemoglobinuria)	237 (4.4)	121 (51.1)	116 (48.9)
Severe jaundice	114 (2.1)	55 (48.3)	59 (51.8)
Anuria / Oliguria (in adults and children—History)	7 (0.1)	6 (85.7)	1 (14.3)
Hypoglycaemia (MB-Stix < 3 mmol/L or clinical improvement after iv glucose)	557 (10.3)	278 (49.9)	279 (50.1)
Hb g/dl: Mean: 6.5, IQR (4.8–8.8)	6.5 (4.6–8.8)	6.8 (4.6–8.8)	
BUN mg/dl: Mean 13, IQR: 9–20)	13 ( 9−21)	13 (9 –20)	
Base excess: Mean − 8.78, IQR:—13 to − 4)	– 7 (−13 to − 4)	– 8 (−8 to − 4)	

### Prognostic factors of metabolic acidosis

In this study, 50.1% (2,296) of children presented with severe metabolic acidosis [base excess less than 8 mmol/l] and there was no significant difference between males (48.9%) and females (51.3%). About a quarter, 24.1% had normal acid–base state and 25.8% had mild metabolic acidosis (BE: more than 8 mmol/l and less than 3 mmol/l). Among those who died, 82% (367), had severe metabolic acidosis. Survivors were 81% were less likely to have severe metabolic acidosis compared to those who died (OR = 0.19, 95% CI 0.15–0.24, p-value: < 0.001). Severe metabolic acidosis was present in 50.8% (1162) of patients in the artesunate arm and 49.5% (1134) of children in the quinine arm.

For the multivariable analysis (Table 2), every year increase in age was associated with a 21% decrease in likelihood of presenting with severe metabolic acidosis (OR: 0.79, CI 0.76–0.83).

**Table 2:** Univariable and multivariable analysis of admission features and their association with severe metabolic acidosis

Variable	Sample size: 5425		Univariable analysis			Multivariable analysis		
	N (%)	Severe Metabolic Acidosis N (%)	Odds ratio	95% CI	P-Value	Odds ratio	95% CI	P Value
Gender: female (reference) and male	2611 (48.1)	1143 (51.3)						
	2815 (51.9)	1153 (48.9)	0.91	0.81–1.02	0.11	0.92	0.19–4.55	0.92
Age	5426		0.78	0.76–0.81	< 0.001	0.79	0.76–0.83	< 0.001
Coma (BCS < = 2)	1823 (33.6)	776 (50.6)	1.03	0.91–1.16	0.646	1.72	1.17–2.51	0.005
Prostration	2974 (54.8)	1299 (52.3)	1.21	1.07–1.36	0.002	1.88	1.35–2.59	< 0.001
Convulsions (> 30 min)	169 2(31.2)	623 (43.4)	0.68	0.60–0.77	< 0.001	0.7	0.53–0.93	0.012
Decompensated shock: systolic BP < 70mhg	178 (3.3)	110 (71.9)	2.62	1.84–3.75	< 0.001	1.46	0.66–3.32	0.352
Respiratory distress	867 (15.9)	498 (67.8)	2.39	2.03–2.83	< 0.001	1.46	1.02–2.1	0.039
Deep breathing	938 (17.3)	731 (86.3)	8.74	7.11 –10.74	< 0.001	3.94	2.51–6.2	< 0.001
Black water fever (haemoglobinuria)	237 (4.4)	73 (40.6)	0.67	0.49–0.91	0.009	1.28	0.78–2.11	0.34
Symptomatic anaemia	114 (2.1)	55 (56.12)	1.28	0.86–1.92	0.23			
Anuria / Oliguria	7 (0.1)	7 (100)	1	*	*			
Hyperparasitaemia	100 (1.8)	40 (42.6)	0.73	0.48–1.11	0.14			
Mild anaemia (< 10—5)	2705 (55.0)	1139 (45.3)	1.21	1.02–1.43	0.027	0.73	0.54–0.98	0.04
Severe anaemia (< 5)	1431 (29.1)	841 (64.2)	2.6	2.16–3.13	< 0.001	0.79	0.55–1.12	0.19
Uraemia (BUN > 20)	1110 (24.3)	800 (73.4)	3.55	3.05-–4.13	< 0.001			
Hypoglycaemia	557 (10.3)	434 (88.4)	9.11	6.87–12.09	< 0.001	5.16	2.74–9.75	< 0.001

Respiratory distress = costal in drawing/ recession / respiratory insufficiency; Hyperparasitaemia =  > 500 Parasites per high power field

The clinical features with the strongest association to severe metabolic acidosis were deep breathing (OR: 3.94, CI 2.51–6.2) and hypoglycaemia (OR: 5.16, CI 2.74–9.75).

In the multivariable analysis, children presenting with a coma were 1.72 times more likely to have severe metabolic acidosis when compared to those without coma (CI 1.17–2.51). Conversely, children who presented with convulsions were 30% less likely to have severe metabolic acidosis compared to those without convulsions (OR: 0.7, CI 0.53–0.93).

Children with respiratory distress (OR: 1.46, CI 1.02–2.1) and prostration (OR: 1.88 CI 1.35–2.59) more likely to have metabolic acidosis compared to those who did not have these signs.

**Fig. 2:**
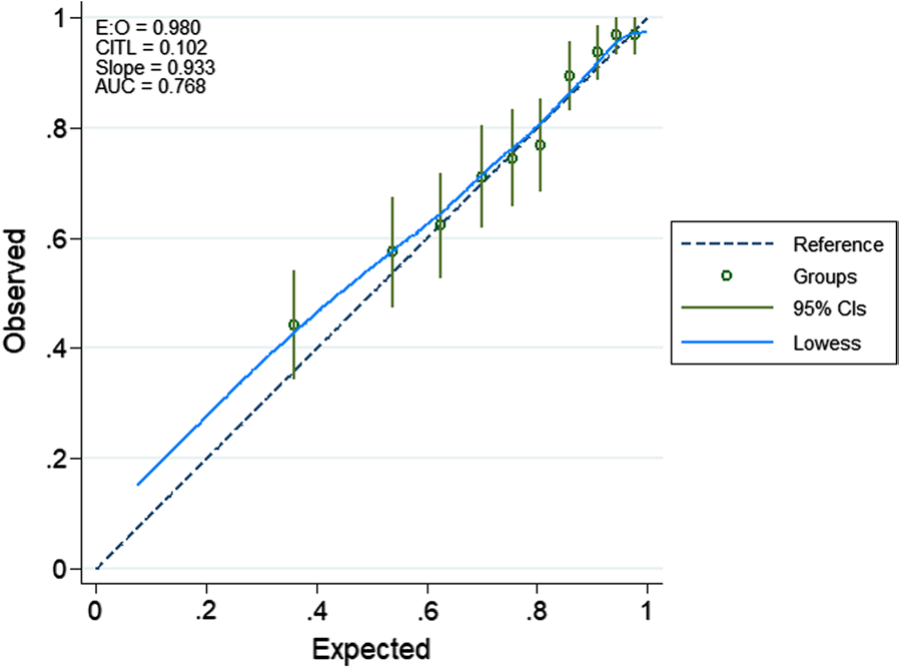
Calibration plot of severe metabolic acidosis model

Model performance is shown using a calibration plot in Fig. 2 of observed against predicted events plotted for 10 risk groups (c-index = 0.76, slope = 0.9). The Area Under the Curve (AUC) (Fig. 2) based on the final model was 0.77. The model has a positive predictive value (PPV) of 88.7% and a Negative Predictive value (NPV) of 43.1%. The sensitivity was 67.3% and the specificity was 74.2%. The model had internal validation performed using bootstrapping with 5000 simulations resulting in AUC of 0.78 (95% CI 0.76–0.79).

### Prognostic factors of uraemia

Among children admitted with severe malaria in this study, 24.3% (1110) had uraemia. Uraemia was present among 217 participants (49.2%) out of 441 who died (missing data on BUN for 86 results) and of the 4120 participants who survived, 21.7% (893) had Uraemia. Those who survived were 71% less likely to have uraemia compared to those who died (OR: 0.29, CI 0.23–0.35, p-value: < 0.001). The univariable analysis for factors associated with uraemia are shown in the Table 3.

**Table 3:** Univariable and multivariable analysis of clinical features associated with uraemia at admission

Variable	Sample size: 5425		Univariable analysis			Multivariable analysis		
	N (%)	Acute kidney injury N(%)	Odds Ratio	CI 95%	P-value	Odds Ratio	CI 95%	P-value
Age (Mean: 2 years IQR:25th -75th: 1–4)			1.04	1.01–1.07	0.003	1.08	1.05—1.13	< 0.001
Gender: female (reference), male	2611 (48.1)	533 (24.1)	1.03	0.89—1.18	0.688	1	0.86—1.16	0.97
Coma (BCS < = 2)	1823 (33.6)	436 (28.8)	1.42	1.23–1.63	< 0.001	3.18	2.36—4.27	< 0.001
Severe prostration	2974 (54.8)	574 (23.1)	0.86	0.75–0.99	0.034	1.78	1.37—2.30	< 0.001
Convulsions (> 30 min)	1692 (31.2)	293 (20.6)	0.74	0.63–0.86	< 0.001	0.83	0.68—1.02	0.075
Decompensated shock	178 (3.3)	59 (39.3)	2.07	1.48–2.89	< 0.001	1.89	1.31—2.74	0.001
Respiratory distress	867 (15.9)	198 (27.1)	1.19	0.99–1.43	0.056	1.1	0.89—1.35	0.366
Deep breathing	938 (17.3)	255 (30.9)	1.5	1.27–1.78	< 0.001	2.05	1.65—2.55	< 0.001
Black water fever (haemoglobinuria)	237 (4.4)	58 (32.0)	1.49	1.08—2.05	0.014	1.58	1.09—2.27	0.014
Severe jaundice	114 (2.1)	49 (50)	3.21	2.15– 4.79	< 0.001	3.46	2.21- 5.43	< 0.001
Anuria /Oliguria	7 (0.1)	6 (85.7)	18.75	2.25–155.9	0.007			
Hyperparasitaemia	100 (1.8)	14 (15.6)	0.57	0.32 –1.01	0.053			
Mild anaemia (< 10 −5)	2705 (55.0)	593 (23.6)	1.54	1.24- 1.91	< 0.001	1.28	1.01—1.62	0.04
severe anaemia (> 5)	1431 (29.1)	393 (29.9)	2.13	1.69–2.67	< 0.001	1.77	1.36—2.29	< 0.001
Base excess: More than -8 mmol/L (reference)	2286 (49.9)	290 (12.9)						
Severe metabolic acidosis	2296 (50.1)	800 (35.6)	3.73	3.21 – 4.33	< 0.001			
Blood Glucose: hypoglycaemia: < 3 mmol/L), No Hypoglycaemia (Reference)	557 (10.3)	223 (45.7)	3.02	2.49–3.67	< 0.001	2.77	2.22—3.46	< 0.001

Severe Prostration: Not Able to breast feed < 6 m or able to sit > 6 m, Decompensated Shock: Children Systolic BP < 70mhg, Respiratory distress: costal in drawing/ recession / respiratory insufficiency, Hyperparasitaemia: > 500 parasites per high powered field

After multivariable analysis (Table 3) and forward selection of predictors, factors associated with uraemia upon admission were; increasing age (OR: 1.08, CI 1.05–1.13), coma (OR: 3.18, CI 2.36–4.27), prostration (OR: 1.78 CI 1.37–2.30), decompensated shock (OR: 1.89, CI 1.31–2.74), black water fever (OR: 1.58, CI 1.09–2.27), jaundice (OR: 3.46 CI 2.21–5.43), mild anaemia (OR: 1.28, CI 1.01–1.62), severe anaemia (OR: 1.77, CI 1.36–2.29) and hypoglycaemia (OR: 2.77, CI 2.22–3.46).

**Fig. 3:**
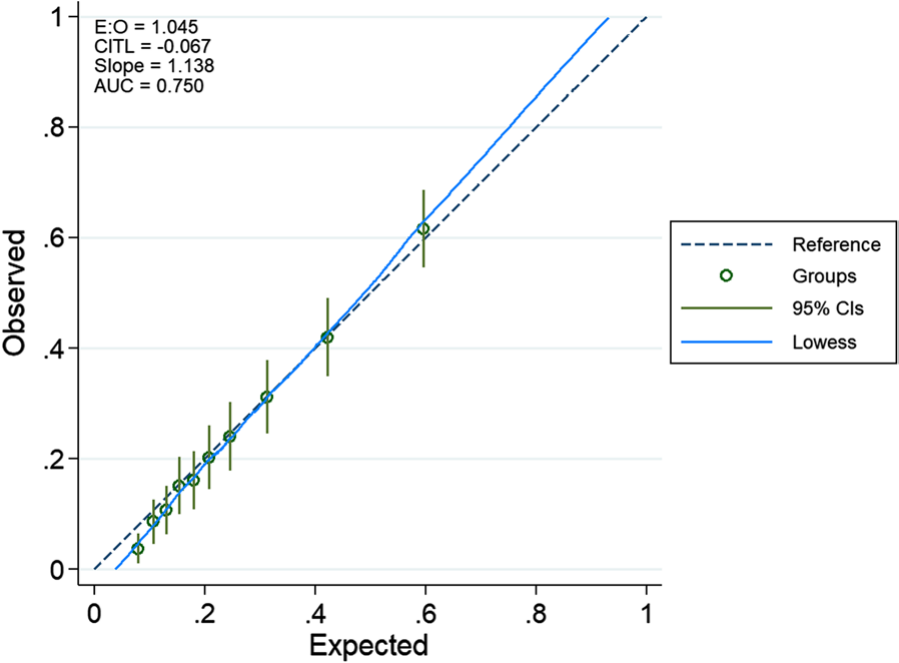
Calibration plot of uraemia model

The model performance was assessed using a calibration plot (Fig. 3) of observed against predicted events for 10 groups and produced a c-index = 0.75, slope = 1.1). The AUC ROC for the final model was 0.74. The sensitivity was 67.4% and the specificity was 67.4%. The final model had a PPV of 39.9%, an NPV of 86.5% and correctly classified 67.4% of the values.

The model had an internal validation performance of AUC = 0.74 (95% CI 0.72–0.76) using bootstrapping with 5000 simulations.

## Discussion

Although severe metabolic acidosis and acute kidney injury are major causes of death from severe malaria, diagnosing them is challenging in low resource settings. This study identified clinical features of severe metabolic acidosis and uraemia in children with severe malaria at admission. The models described can be used to predict a group of children with a high chance of having severe metabolic acidosis or acute kidney injury, which could then be selected for further laboratory work-up. Diagnosing these complications is important for the clinical management, monitoring, and, in the case of AKI, deciding on potential kidney replacement therapy.

### Severe metabolic acidosis

In this study, the clinical features associated with severe metabolic acidosis in children with severe falciparum malaria at admission were young age and prostration at admission. Clinical features that contributed to severe metabolic acidosis were severe anaemia, hypoglycaemia, acute kidney injury and black water fever. Features occurring because of severe metabolic acidosis were respiratory distress, deep breathing and coma at admission. All signs can be used to identify the group with a high probability of having severe metabolic acidosis.

Similar findings were identified in a prognostic study in The Gambia with discriminative performance (ROC: 0.83) [17]. This study identified; low Blantyre coma score (BCS: 0–2), high parasitaemia, high respiratory rate for age and deep breathing as clinical features associated with hyperlactataemia in children with severe malaria [17]. Hyperparasitaemia was not associated with severe metabolic acidosis in this study and, this may be attributed to the study in The Gambia occurring in a low transmission setting.

Signs of respiratory distress are commonly associated with severe metabolic acidosis, as is the finding in this study and studies in The Gambia, Malawi and Kenya [19, 24, 26, 32]. Despite the potential overlap between respiratory distress from severe malaria and severe pneumonia, the data suggest that respiratory distress is a major independent predictor of severe metabolic acidosis [33–35].

This study suggests that deep breathing is a strong independent predictor of severe metabolic acidosis as it is an important element of respiratory compensation as a consequence of metabolic acidosis [36]. In this study, children with severe malaria presenting with deep breathing were 8 times more likely to have severe metabolic acidosis upon univariable analysis (OR: 8.74, CI 7.11 -10.74). Although inter-observer variability may affect the use of this sign as a surrogate marker for severe metabolic acidosis, it may be a reliable starting point for screening for children who require specialized management upon admission [37].

In this study, younger children were more likely to have Severe Metabolic Acidosis upon admission with severe malaria. Although a study in South East Asia found that metabolic acidosis did not vary with age, the findings from this study are consistent with clinical trials in Tanzania [2, 38]. This may suggest that the higher transmission rates of malaria in Africa increase the odds of severe metabolic acidosis in children.

Mtove et al. found that severe anaemia and hypoglycaemia were significantly associated with hyperlactataemia upon univariable logistic regression, as was the case in this study [38]. Severe anaemia is a known complication of malaria and contributes to fluid depletion and hypoxia in children with severe malaria and, hypoglycaemia is a major cause of mortality in children with malaria [9]. These are complications of malaria that contribute to causing severe metabolic acidosis.

In contrast with this study, Newton et al*.* found deep breathing, coma, and hypoglycaemia to be highly specific for hyperlactatemia in three different countries [20]. Their study concluded that these were not reliable surrogate markers of severe metabolic acidosis [20].

The model generated in this study performed well and, upon internal validation, deep breathing, hypoglycaemia, and coma were reliable prognostic predictors of severe metabolic acidosis. Prospective, external validation studies are recommended to explore the generalizability of this model. To contribute to the improvement of the clinical management of these complications, further studies should study the effect of blood transfusions, fluid management and oxygen support to children identified as severe metabolic acidosis.

### Uraemia

Older children and children who presented with coma, jaundice and hypoglycaemia were more likely to be uraemic upon admission with severe malaria in this analysis. Children who presented with decompensated shock, blackwater fever, anaemia (mild and severe), anuria and severe metabolic acidosis were also more likely to have uraemia.

AKI is often underdiagnosed in children, and an observed prevalence of uraemia, of 24%, among all severe malaria admissions and 49% among all children who died in this study is alarming. A study in the Gambia found that 25% of children with cerebral malaria had a glomerulo-tubular pattern of kidney pathology [39]. Although that study lacked the power to suggest a significant association between mortality and AKI in severe malaria, it is consistent with the strong significance of coma as an associated feature to AKI in this study [39].

In this study, only 7 children had oliguria (out of 5426) and all of them had uraemia. This may reflect inconsistencies in defining oliguria, as this variable was not objectively measured through catheterization. However, absence of oliguria does not exclude kidney injury one study found that 75% of patients with acute kidney failure secondary to severe malaria were non-oliguric [40].

While black water fever was significantly associated with uraemia, it was present in only 237 of 5426 children (4.4%) [4]. Children with severe falciparum malaria in India had an AKI incidence of 19% though none had black water fever [41]. In contrast, black water fever was the leading cause of AKI among children in a nephrology unit in the Democratic Republic of Congo [12]. Additionally, in Uganda AKI was associated with haemoglobinuria in children cerebral malaria and severe malaria anaemia [23].

The findings of anaemia (mild and severe) and decompensated shock being significantly associated with AKI are consistent with studies that identify the role of dehydration in the development of AKI in severe malaria [12, 39, 41]. Further study is needed to understand appropriate fluid management in children as fluid bolus resuscitation was associated with increased mortality in a large trial [42].

The analysis from this study suggests that severe metabolic acidosis and uraemia are interrelated. Hypoglycaemia, coma and severe anaemia are features common to these two outcomes. This could reinforce these as major targets for clinical interventions. In addition to supportive care, patients would require rehydration with intravenous fluids, but not with fluid bolus therapy as discussed in the FEAST study [42]. Dialysis could be started early if AKI is confirmed, and further study is needed to guide dialysis for treatment of severe metabolic acidosis irrespective of concomitant kidney failure.

Increasing age was significantly associated with uraemia in this study. Similarly, a higher mean age of kidney involvement in children with severe malaria was seen in the DRC (6.7 years), India (7.7 years) and south-east Asia [2, 12, 41]. This was attributed to a delay of acquired immunity and increase of severe malaria in older children as malaria prevention efforts intensified for younger children over the years. This may be on on-going trend, as an observational study in Kenya found that between 1989 and 2016, there was a marked increase in the mean age of children presenting with cerebral malaria and in those who died from malaria related causes, when compared to those presenting without malaria related conditions [33].

Although the model generated in this study can be used in these settings, it may also be useful to invest in biomarker diagnoses for AKI in African hospitals. An example is the saliva urea nitrogen dipstick test that has been shown to be useful in identifying AKI in children and adults with severe malaria [43, 44].

## Limitations

A major limitation was the inability to follow up the clinical progress of severe metabolic acidosis or uraemia for the study participants bacause this study was a retrospective analysis. There is a risk of inter-observer variation in identifying clinical signs which may over or under emphasize the presence of some features. Secondly, there is possible collinearity between uraemia and severe metabolic acidosis as there were clinical factors common to both outcomes. However, the analysis in this study was unable to explore the implications of the collinearity on presentation at admission or prognosis in children with severe malaria. A previously developed model for severe metabolic acidosis [38], could not be tested due to differences in how severe metabolic acidosis was measured. Despite this, the performance of both models and the clinical factors identified to be associated with severe metabolic acidosis, were similar to those in this study. Lastly, studying kidney injury was limited to analysing blood urea nitrogen levels. Creatinine and urine output were not available for this analysis and AKI could not be defined by KIDGO standards.

## Conclusions

A secondary analysis was conducted of a large clinical trial spanning nine African countries to identify prognostic features of severe metabolic acidosis and uraemia in children with severe malaria. Identifying children who are likely to have these complications could help triage patients requiring further treatment or specialized therapy. Health policies should consider structures for early identification and treatment of these complications to reduce mortality from malaria.

## Data Availability

The datasets used and/or analysed during the current study are available from the corresponding author on reasonable request.

## Abbreviations

AQUAMAT: Artesunate vs Quinine in the treatment of severe falciparum malaria in African children.
PPV: Positive Predictive Value
NPV: Negative Predictive Value
AUC: Area Under the Curve
ROC: Receiver Operating Characteristic
WHO: World Health Organization
AKI: Acute Kidney Injury
αHBA: α-Hydroxybutyric acid
βHBA: β-Hydroxybutyric acid
HPLA: P-hydroxyphenyllactic acid
BUN: Blood Urea Nitrogen
BE: Base Excess
OR: Odds Ratio
CI: Confidence Interval
Hb: Haemoglobin
VIF: Variance Inflation Factor
BWF: Black Water Fever
BCS: Blantyre Coma Score

## References

[1] World malaria report 2018. Geneva: World Health Organization; 2018. Licence: CC BY-NC-SA 3.0 IGO. https://www.who.int/publications/i/item/9789241565653. Accessed 11 July 2019.

[2] Dondorp AM, Lee SJ, Faiz MA, Mishra S, Price R, Tjitra E (2008). The relationship between age and the manifestations of and mortality associated with severe malaria. Clin Infect Dis.

[3] Kazembe LN, Kleinschmidt I, Sharp BL (2006). Patterns of malaria-related hospital admissions and mortality among Malawian children : an example of spatial modelling of hospital register data. Malar J.

[4] Von SL, Olaosebikan R, Hendriksen ICE, Lee SJ, Adedoyin OT, Agbenyega T (2012). Predicting the clinical outcome of severe falciparum malaria in African children : findings from a large randomized trial. Clin Infect Dis.

[5] Leopold SJ. Metabolomic characterization of acidosis in severe falciparum malaria. DPhil Thesis, University of Oxford; 2018. https://ora.ox.ac.uk/objects/uuid:3a089f85-39dc-478f-b627-37accb5004ea. Accessed 27 July 2019.

[6] Sriboonvorakul N, Ghose A, Hassan MMU, Hossain A, Faiz MA, Pukrittayakamee S (2018). Acidosis and acute kidney injury in severe malaria. Malar J.

[7] Herdman MT, Sriboonvorakul N, Leopold SJ, Douthwaite S, Mohanty S, Hassan MMU (2015). The role of previously unmeasured organic acids in the pathogenesis of severe malaria. Crit Care.

[8] White NJ, Pukrittayakamee S, Hien TT, Faiz MA, Mokuolu OA, Dondorp AM (2018). Malaria. Lancet.

[9] Maitland K, Newton CRJC (2005). Acidosis of severe falciparum malaria: heading for a shock?. Trends Parasitol.

[10] Patel H, Dunican C, Cunnington AJ (2020). Predictors of outcome in childhood *Plasmodium falciparum* malaria. Virulence.

[11] Trang TT, Phu NH, Vinh H, Hien TT, Cuong BM, Chau TT (1992). Acute renal failure in patients with severe falciparum malaria. Clin Infect Dis.

[12] Aloni MN, Nsibu CN, Meeko-Mimaniye M, Ekulu PM, Bodi JM (2012). Acute renal failure in Congolese children: a tertiary institution experience. Acta Paediatr.

[13] Esezobor CI, Ladapo TA, Osinaike B, Lesi FEA (2012). Paediatric acute kidney injury in a tertiary hospital in Nigeria: prevalence, causes and mortality rate. PLoS ONE.

[14] Maitland K, Kevin M, English M, Mithwani S, Peshu N, Marsh K (2003). Severe P falciparum malaria in Kenyan children : evidence for hypovolaemia. QJM..

[15] Marsh K, Forster D, Waruiru C, Mwangi I, Winstanley M, Marsh V (2019). Indicators of life-threatening malaria in african children. N Engl J Med.

[16] Dondorp AM, Chau TTH, Nguyen HP, Mai NTH, Pham PL, Ly VC (2004). Unidentified acids of strong prognostic significance in severe malaria. Crit Care Med.

[17] Bhaskaran K, Ebonyi AO, Walther B, Walther M (2013). Predictors of hyperlactataemia among children presenting with malaria in a low transmission area in The Gambia. Malar J.

[18] Crawley J, English M, Waruiru C, Mwangi I, Marsh K (1998). Abnormal respiratory patterns in childhood cerebral malaria. Trans R Soc Trop Med Hyg.

[19] Taylor TE, Borgstein A, Molyneux ME (1993). Acid-base status in paediatric *Plasmodium falciparum* malaria. Q J Med.

[20] Newton CRJC, Valim C, Krishna S, Wypij D, Olola C, Agbenyega T (2005). The prognostic value of measures of acid/base balance in pediatric falciparum malaria, compared with other clinical and laboratory measures. Clin Infect Dis.

[21] Conroy AL, Hawkes M, Elphinstone RE, Morgan C, Hermann L, Barker KR (2016). Acute kidney injury is common in pediatric severe malaria and is associated with increased mortality. Open Forum Infect Dis.

[22] Olowu WA, Niang A, Osafo C, Ashuntantang G, Arogundade FA, Porter J (2016). Outcomes of acute kidney injury in children and adults in sub-Saharan Africa: a systematic review. Lancet Glob Health.

[23] Conroy AL, Opoka RO, Bangirana P, Idro R, Ssenkusu JM, Datta D (2019). Acute kidney injury is associated with impaired cognition and chronic kidney disease in a prospective cohort of children with severe malaria. BMC Med.

[24] English M, Waruiru C, Amukoye E, Murphy S, Crawley J, Mwangi I (1996). Deep breathing in children with severe malaria : indicator of metabolic acidosis and poor outcome. Am J Trop Med Hyg.

[25] Dondorp AM, Fanello CI, Hendriksen IC, Gomes E, Seni A, Chhaganlal KD (2010). Artesunate versus quinine in the treatment of severe falciparum malaria in African children (AQUAMAT): an open-label, randomised trial. Lancet.

[26] English M, Sauerwein R, Waruiru C, Mosobo M, Obiero J, Lowe B (2002). Acidosis in severe childhood malaria. QJM.

[27] Plewes K, Turner GDH, Dondorp AM (2018). Pathophysiology, clinical presentation, and treatment of coma and acute kidney injury complicating falciparum malaria. Curr Opin Infect Dis.

[28] Chellappan A, Bhadauria DS (2016). Acute kidney injury in malaria: an update. Clin Queries Nephrol.

[29] Barros Pinto MP, Marques G (2020). Severe malaria. Trop Med Int Health.

[30] Khwaja A (2012). KDIGO Clinical practice guidelines for acute kidney injury. Nephron Clin Pract.

[31] Textor J, van der Zander B, Gilthorpe MS, Liskiewicz M, Ellison GT (2017). Robust Causal Inference using directed acyclic graphs: the R package dagitty. Int J Epidemiol.

[32] Jallow M, Casals-Pascual C, Ackerman H, Walther B, Walther M, Pinder M (2012). Clinical features of severe malaria associated with death : a 13-year observational study in The Gambia. PLos ONE.

[33] Njuguna P, Maitland K, Nyaguara A, Mwanga D, Mogeni P, Mturi N (2019). Observational study: 27 years of severe malaria surveillance in Kilifi. Kenya BMC Med.

[34] O’Dempsey TJD, McArdle TF, Laurence BE, Todd JE, Greenwood BM, Lamont AC (1993). Overlap in the clinical features of pneumonia and malaria in african children. Trans R Soc Trop Med Hyg.

[35] English M, Punt J, Mwangi I, McHugh K, Marsh K (1996). Clinical overlap between malaria and severe pneumonia in African children in hospital. Trans R Soc Trop Med Hyg.

[36] Marsh K, Forster D, Waruiru C, Mwangi I, Winstanley M, Marsh V (1995). Indicators of life-threatening malaria in african children. N Engl J Med.

[37] English M, Murphy S, Mwangi I, Crawley J, Peshu N, Marsh K (1995). Interobserver variation in respiratory signs of severe malaria. Arch Dis Child.

[38] Mtove G, Nadjm B, Hendriksen ICE, Amos B, Muro F, Todd J (2011). Point-of-care measurement of blood lactate in children admitted with febrile illness to an African district hospital. Clin Infect Dis.

[39] Weber MW, Zimmermann U, Van HMB, Frenkel J, Palmer A, Ehrich JHH (1999). Renal involvement in Gambian children with cerebral or mild malaria. Trop Med Int Health.

[40] Gupta BK, Nayak KC, Kumar S, Kumar S, Gupta A, Prakash P (2012). Oliguric and non-oliguric acute renal failure in malaria in west zone of Rajasthan, India: a comparative study. J Acute Dis.

[41] Prasad R, Mishra OP (2016). Acute kidney injury in children with *Plasmodium falciparum* malaria: Determinants for mortality. Perit Dial Int.

[42] Maitland K, Kiguli S, Opoka RO, Engoru C, Olupot-Olupot P, Akech SO (2011). Mortality after fluid bolus in African children with severe infection. N Engl J Med.

[43] Hussein RH, Calice-Silva V, Evans R, Levin NW, Ellidir RA, Ali EM (2020). Diagnosis of acute kidney injury in children hospitalized in a sub-Saharan African unit by saliva urea nitrogen dipstick test. Blood Purif.

[44] Calice-Silva V, Sacomboio E, Raimann JG, Evans R, Dos Santos SC, Tchivango AT (2018). Diagnostic performance of salivary urea nitrogen dipstick to detect and monitor acute kidney disease in patients with malaria. Malar J.

